# 

**Published:** 1877-05

**Authors:** 


MAY, 1877.

Lot-132

### THE AMERICAN JOURNAL OF DENTAL SCIENCE,

EDITED BY

F. J. S. GORGAS, M. D., D. D. S.

PUBLISHED MONTHLY.

$2.50 PER ANNUM.

VOL. 11.—THIRD SERIES.—No. 1.

BALTIMORE:

SNOWDEN & COWMAN.

LONDON:

TRUBNER & CO., 60 PATERNOSTER ROW.

1877.

PAYABLE IN ADVANCE.

